# Deep Dual-Resolution Road Scene Segmentation Networks Based on Decoupled Dynamic Filter and Squeeze–Excitation Module

**DOI:** 10.3390/s23167140

**Published:** 2023-08-12

**Authors:** Hongyin Ni, Shan Jiang

**Affiliations:** 1 School of Computer Science, Northeast Electric Power University, Jilin 132012, China; 2 Gongqing Institute of Science and Technology, No. 1 Gongqing Road, Gongqing 332020, China

**Keywords:** road scene segmentation, deep dual-resolution networks, squeeze-and-excitation, decoupled dynamic filter

## Abstract

Image semantic segmentation is an important part of automatic driving assistance technology. The complexity of road scenes and the real-time requirements of application scenes for segmentation algorithm are the challenges facing segmentation algorithms. In order to meet the above challenges, Deep Dual-resolution Road Scene Segmentation Networks based on Decoupled Dynamic Filter and Squeeze–Excitation (DDF&SE-DDRNet) are proposed in this paper. The proposed DDF&SE-DDRNet uses decoupled dynamic filter in each module to reduce the number of network parameters and enable the network to dynamically adjust the weight of each convolution kernel. We add the Squeeze-and-Excitation module to each module of DDF&SE-DDRNet so that the local feature map in the network can obtain global features to reduce the impact of image local interference on the segmentation result. The experimental results on the Cityscapes dataset show that the segmentation accuracy of DDF&SE-DDRNet is at least 2% higher than that of existing algorithms. Moreover, DDF&SE-DDRNet also has satisfactory inferring speed.

## 1. Introduction

Automatic driving assistance technology ensures driving safety and smooth traffic from different perspectives, such as driving strategies [1] and target detection [2,3]. Target detection algorithms such as YOLOv3 [4] perform well in target detection tasks with fixed shapes in road scenes, and can frame objects with fixed shapes such as vehicles or pedestrians, but for objects without shapes, such as the sky or a road, target detection is invalid. Road scene segmentation segments different objects in the road scene image, which can correctly obtain the semantic information of the road scene. This technology can provide basic data support for unmanned driving and intelligent assisted driving [5].

The performance of deep learning methods in semantic segmentation [6] has exceeded that of traditional methods. Most image semantic segmentation networks [7,8] based on deep learning methods are based on Fully Convolutional Networks (FCNs) [9] proposed by Long et al.

According to different types of roads, road scenes include urban streets, highways, rural roads, etc. Depending on the weather, road scenes include rain, snow, sunny days, or heavy fog. There are many miscellaneous objects in road scenes, including pedestrians, vehicles, and objects of different sizes. The complexity of road scenes brings great challenges to the segmentation of road scenes. As an important part of automatic assisted driving, road scene segmentation algorithms require high real-time performance and low computation. Because road scene segmentation is an intensive prediction task, the segmentation network needs to output a high-resolution feature map, which conflicts with the real-time requirements of the segmentation task.

At present, there are many lightweight and real-time image semantic segmentation networks. BiseNet [10] is a lightweight semantic segmentation network that includes spatial and contextual paths for obtaining image features. Du et al. [11] designed an urban road scene segmentation network based on BiseNet. This network solves the problem of losing some pixel feature information caused by continuous down-sampling operations in the Bisenet network. However, the experimental results of this network on the Cityscapes dataset are not ideal. Kherraki et al. [12] believe that accuracy is not the first consideration in road scene segmentation tasks. They proposed a new network based on an encoder–decoder architecture, which reduces accuracy with fewer parameters. However, this method sacrifices too much accuracy for speed. Paszke et al. [13] proposed Efficient Neural Network (ENet), which is 18 times faster than SegNet [14]. However, many segmentation networks based on deep learning will first reduce the resolution of sample image features in the feature extraction stage and then restore the resolution of features through an upsampling operation. Such an approach will cause the loss of spatial information in the sample image. To solve this problem, Hong et al. [15] proposed deep dual-resolution networks (DDRNets). DDRNet has two branches: the upper branch is a low-resolution branch and the lower branch is a high-resolution branch. Each branch of DDRNet consists of multiple sequential residual basic blocks (RBs) and residual bottleneck block (RBB) modules. DDRNet has a satisfactory segmentation effect and high real-time performance. DDRNet has three network versions: DDRNet-23slim, DDRNet-23 and DDRNet-39.

Although there are many studies demonstrating excellent accuracy and real-time performance, further research is still needed to overcome the challenges introduced by the complexity of the road scene and further improve the real-time performance of the segmented network. This paper proposed a road scene segmentation network based on Decoupled Dynamic Filter and Squeeze and Excitation (DDF&SE-DDRNet). The proposed DDF&SE-DDRNet uses a decoupled dynamic filter [16] to replace the ordinary convolution of RB and RBB in DDRNet, and adds a squeeze and excitation module (SE-Module) [17] to each module of the network. The main contributions of this paper are as follows:

(1) DDF is used to replace the ordinary convolution of each module in the network, which reduces the number of network parameters, and enables the network to dynamically adjust the weight of each convolution kernel according to the sample image.
(2) By adding SE-Module to each module in the network, the local feature map in the network can obtain global features, which reduces the impact of image local interference on the segmentation effect.
(3) We conducted experiments on the Cityscapes dataset to evaluate the proposed DDF&SE-DDRNet. The experimental results show that the performance of the proposed DDF&SE-DDRNet is better than that of the state of the art.

The rest of the paper is organized as follows. Section 2 gives a brief introduction to Deep Dual-resolution Networks (DDRNet), decoupled dynamic filters, and Squeeze-and-Excitation. Section 3 introduces the proposed DDF&SE-DDRNet in detail. Section 4 proves the effectiveness of the proposed DDF&SE-DDRNet through experiments. The last part concludes the paper.

## 2. Related Work

### 2.1. Deep Dual-Resolution Networks

**Figure 1:**
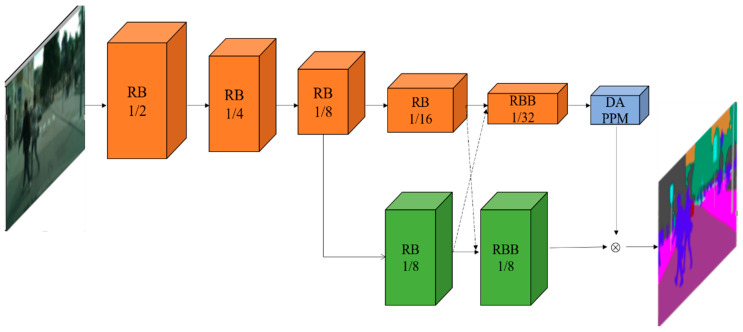
Architecture of DDRNet.

Figure 1 shows the network structure of DDRNet. In Figure 1, the orange branch extracts rich context information through multiple downsampling operations, and the other green branch can generate a relatively high-resolution feature map.

DDRNet consists of multiple sequential residual basic blocks (RBs) and residual bottleneck block (RBB) modules. The DAPPM denotes the Deep Aggregation Pyramid Pooling Module. In DAPPM, the context extracted from larger pooled cores is integrated with deeper information flow, and multi-scale characteristics are formed by integrating pool cores with different depths and sizes. DDRNet is divided into five network stages. The first to third stages are, respectively, composed of three RB modules of the upper branch. The fourth stage consists of the fourth orange RB module of the upper branch and the first green RB module of the lower branch. The fifth stage consists of the orange RBB module of the upper branch and the green RBB module of the lower branch. The black dotted line represents the Bilateral fusion operation.

The input of DDRNet is a 224 × 224-pixel image. In the low-resolution branch of DDRNet, the size of the feature map will be reduced by half every time it passes through a network stage. After passing through five RB modules and one RBB module, the size of the output feature map is 7 × 7. The high-resolution branch uses the output of the third RB module of the low-resolution branch, and the characteristic figure is maintained in the next two modules 28 × 28 size unchanged to extract high-resolution feature information.

### 2.2. Attention Mechanism Based on Squeeze-and-Excitation

The visual attention mechanism has proven to be better at extracting features from images [18]. The Squeeze and Exception (SE) visual attention mechanism can recalibrate the features in the network. Through this mechanism, the local features in the network can perceive global information and selectively emphasize the useful information. Suppose the input of the network is $X\in R^{W^{\prime }\times H^{\prime }\times C^{\prime }}$, the feature $U\in R^{W\times H\times C}$ is obtained by a series of convolution and other transformations. The SE attention mechanism then recalibrates the feature U through the following three operations.

The first step is the Squeeze operation, which compresses the spatial dimension through the global average pooling (GAP) operation. That is, the two-dimensional feature (H × W) of each channel is compressed into a real number because this real number is calculated based on all the values of the two-dimensional feature of each channel; therefore, it has global information. The number of channels after compression remains unchanged, and the feature U becomes 1 × 1 × C after the Squeeze operation.

The second step is the Excitation operation. Through two fully connected layers, the fully connected layer automatically learns the feature weights according to the loss function and outputs the same weight value as the input feature size.

The third step is the Scale operation, which weighs the normalized weight obtained by the Excitation operation to the features of each channel. Feature recalibration is achieved by multiplying the weight coefficients channel by channel.

### 2.3. Decoupled Dynamic Filter

Decoupled dynamic filter (DDF) is a dynamic filter with two characteristics: it is adaptive and requires less computation than standard convolution. DDF decomposes the traditional convolution operation into a spatial filter branch, a channel filter branch and a short cut connection. This decomposition can greatly reduce the number of parameters and limit the amount of computation to the same level as depth-wise convolution. The spatial filter branch contains a 1 × 1 convolution and a filter norm operation. The channel filter branch has the same structure as the SE-Module. The attention method dynamically adjusts the weight of each convolution kernel according to the input image.

## 3. Approach

### 3.1. Structure of DDF&SE-DDRNet

**Figure 2:**
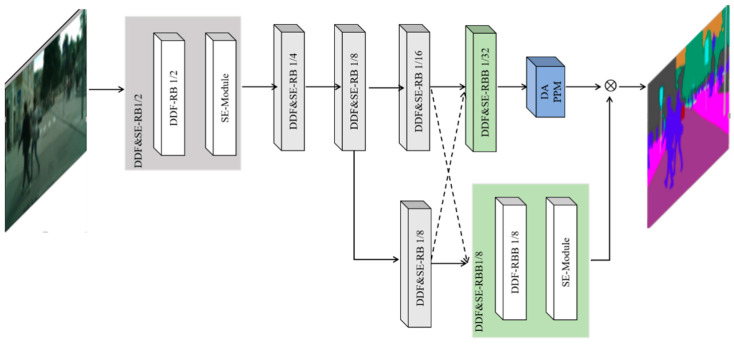
Architecture of DDF&SE-DDRNet.

Figure 2 shows the structure diagram of the proposed DDF&SE-DDRNet. DDF&SE-DDRNet is divided into five network stages. Network stages 1 to 3 consist of DDF&SE-RB1/2, DDF&SE-RB1/4 and DDF&SE-RB1/8 modules of the upper branch. The fourth stage consists of the DDF&SE-RB1/16 module of the upper branch and the DDF&SE-RB1/8 module of the lower branch. The fifth stage consists of the DDF&SE-RBB1/32 modules of the upper branch and the green DDF&SE-RBB1/8 modules of the lower branch. The score in the module name indicates the proportion of the size of the feature map output by the module to the size of the original image. For example, if the size of the original image is 224 × 224, the size of the output feature map of the DDF&SE-RB1/2 module is 112 × 112.

**Figure 3:**
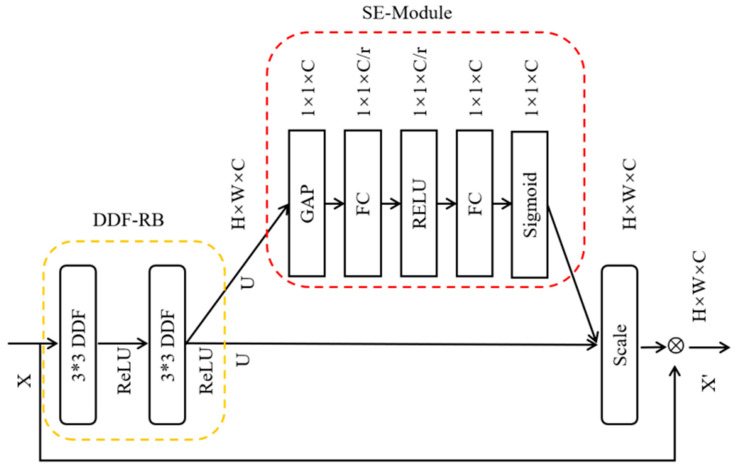
Detailed structure of DDF&SE-RB.

We obtain DDF&SE-RB and DDF&SE-RBB modules by replacing the standard convolutions in the RB and RBB modules in the original network with decoupled dynamic convolutions and adding SE-Modules to these modules. The first grey box in Figure 2 shows the structure of the DDF&SE-RB module. Each DDF&SE-RB module is composed of a DDF-RB module and an SE-Module. Similarly, the green box at the bottom of Figure 2 shows the structure of the DDF&SE-RBB module. The detailed structure of the DDF&SE-RB module is shown in Figure 3.

### 3.2. DDF in the Proposed Network

We replace the ordinary convolution in the RB module in the original network with decoupled dynamic convolution and obtain the DDF-RB module. The yellow dotted box in Figure 3 represents the DDF-RB module. The RB module in the original network is a residual block containing two 3 × 3 convolutional layers. The DDF-RB module consists of two 3 × 3 DDF convolutional layers. Since there is only one 1 × 1 convolutional layer in the spatial filter branch in DDF, the parameter amount of the DDF-RB module is less than that of the RB module. The bottom arrow in Figure 3 is the short cut connection in the original RB module. The RBB module in the original network is also a residual block. The RBB module consists of two 1 × 1 convolutional layers with a 3 × 3 convolutional layer between the two 1 × 1 convolutional layers. The DDF-RBB module uses a DDF convolutional layer to replace the 3 × 3 convolutional layers in the RBB module. If the DDF-RB module in Figure 3 is replaced by the DDF-RBB module, it is the detailed structure diagram of the DDF&SE-RBB module.

### 3.3. SE-Module in the Proposed Network

In the process of acquiring road scene images, due to the influence of factors such as light and the performance of hardware devices, there may be some problems, such as nonuniform light and blur in the collected images. These influencing factors may make the segmentation effect of the segmentation network worse. For example, when local illumination is nonuniform in a road scene, the segmentation network cannot capture enough features from the bright parts. When exposed to rain, snow or foggy weather, the local color and texture blur of the image will affect the feature extraction of the image. When there is local interference in the road scene image, each module of the segmentation network needs information that can reflect the global characteristics of the image to compensate for the influence of the local interference of the image on feature extraction.

The red dotted box in Figure 3 is the structure of the SE-Module in the DDF&SE-RB basic block. In Figure 3, GAP represents global average pooling, FC represents a fully connected layer, Scale represents the feature weight representation layer, and the symbol represents element-wise multiplication. First, the GAP layer compresses the two-dimensional feature (H × W) of each channel in feature map U output by DDF-RB into a real number. Second, the number of feature channels is reduced to 1/r of the number of channels in the feature map U through a fully connected layer. In the third step, after the compressed features are activated by ReLU, the number of channels is raised back to the U dimension through a Fully Connected layer. Using two fully connected layers can make the features have more nonlinear information and can better fit the complex correlation between channels. Next, the feature will obtain the normalized weight between 0 and 1 through a Sigmoid activation function. Finally, a scale operation is used to weigh the normalized weights to each channel of the feature map U. Scale the feature map U before element-by-element multiplication because the channel weight value output by the Sigmoid activation function is a value between 0 and 1. After recalibrating the feature map U with these values, the network is prone to gradient dissipation, and point-by-point multiplication of the recalibrated features with the feature map X transmitted by short cut connection will avoid gradient dissipation.

Although the structure of all SE-Module in the network is the same, the SE-Module plays different roles in different depths. In the shallow layers of the network, the SE-Module enhances the sharing of global information in a class-independent way. In later layers of the network model, the SE-Module responds to different inputs in a highly class-specific way, making the network capability efficient.

## 4. Experiment

We evaluate the proposed DDF&SE-DDRNet on the Cityscapes dataset [19]. The Cityscapes dataset was jointly produced by three German companies, including Daimler, in 2015. The Cityscapes dataset is an image dataset used for a semantic understanding of urban street scenes. In the Cityscapes dataset, there are 5000 street scenes from 50 different cities, with a total of 19 categories of images. The 5000 images are divided into a test set of 1525 images, a training set of 2975 images, and a validation set of 500 images.

### 4.1. Implementation Details

During the training of DDF&SE-DDRNet, we used the SGD optimizer to update the weights of the network model. The size of the epoch is set to 500, the momentum is set to 0.9, the weight decay is set to 0.0005, and the learning rate is set to 0.0001. The CUDA version used in this article is 10.1, the GPU version is NVIDIA Tesla T4×4, and the Ubuntu version is 18.04.6. As an upgraded version of DDRNet, DDF&SE-DDRNet uses the same Loss function as DDRNet.

### 4.2. Evaluation Methodology

In the following experiments, the network performance is mainly evaluated using the PA (Pixel Accuracy) value, MPA (Mean Pixel Accuracy) value, MIou (Mean Intersection over Union) value and the FPS value (Frames Per Second). p_ij_ represents the number of pixels that belong to class i but is predicted to be class j, p_ii_ represents the number of pixels that are predicted to be correct, p_ij_ and p_ji_ are interpreted as false positives and false negatives.

PA represents the proportion of correctly predicted pixels to all pixels. The formula for calculating the PA value is as follows:

(1) $$\mathrm{PA}=\frac{\sum_{i=0}^{k}p_{\mathrm{ii}}}{\sum_{i=0}^{k}\sum_{j=0}^{k}p_{\mathrm{ij}}}$$

mPA calculates the proportion of pixels that are correctly classified in each class, and then averages over all classes. The formula for calculating the mPA value is as follows:

(2) $$\mathrm{mPA}=\frac{1}{k+1}\sum_{i=0}^{k}\frac{p_{\mathrm{ii}}}{\sum_{j=0}^{k}p_{\mathrm{ij}}}$$

However, it is possible that a high number of pixels are correctly divided, but the shape of the segmented area is incorrect. Therefore, MIou is used to measure the shape of the segmented area. MIou computes the mean ratio of union and intersection of ground truth and predicted segmentation. The formula for calculating the MIou value is as follows:

(3) $$\mathrm{MIou}=\frac{1}{k+1}\sum_{i=0}^{k}\frac{p_{\mathrm{ii}}}{\sum_{j=0}^{k}p_{\mathrm{ij}}+\sum_{j=0}^{k}p_{\mathrm{ji}}-p_{\mathrm{ii}}}$$

FPS represents how many frames can be processed every second. The FPS value is higher, the segmentation model is faster.

### 4.3. Road Scene Segmentation on Cityscape Dataset

Table 1 shows the PA value, mPA value, MIou value, FPS value and the number of parameters in the network for the four segmentation methods on the Cityscapes dataset. The four methods in Table 1 are: the original DDRNet-23 as the baseline and our proposed DDF&SE-DDRNet. In addition, Table 1 shows the segmentation effects of the other three state-of-the-art methods, LECNN [12], BiSeNet V2 [20], and ResNet-50 [21].

Table 1 shows the PA value, mPA value, MIou value, FPS value and the number of parameters in the network for the four segmentation methods on the Cityscapes dataset. The four methods in Table 1 are: the original DDRNet-23 as the baseline and our proposed DDF&SE-DDRNet. In addition, Table 1 shows the segmentation effects of the other three state of the art methods. The symbol “-” in Table 1 indicates that the authors of the method do not use PA or mPA to evaluate their proposed method.

From the results in Table 1, we can see that:

(1) DDRNet and DDF&SE-DDRNet outperform BiSeNet V2 and ResNet-50 in both segmentation effect and speed.
(2) The parameter amount of DDF&SE-DDRNet is less than that of the original DDRNet.
(3) The reduction in the number of parameters is the main reason for the improved inferring speed of the DDF&SE-DDRNet road scene segmentation algorithm compared to the original DDRNet road scene segmentation algorithm. The FPS value of the proposed DDF&SE-DDRNet road scene segmentation algorithm is 60, which is higher than the original DDRNet in inferring speed.
(4) Among all networks, LECNN has the highest inference speed and the least number of parameters. However, the MIou value of LECNN is the lowest.
(5) In terms of segmentation accuracy, the PA value, mPA value, and MIou value of the DDF&SE-DDRNet road scene segmentation algorithm are higher than that of LECNN, DDF-DDRNet, and DDRNet.
(6) DDF&SE DDRNet does not sacrifice segmentation accuracy excessively to improve inference speed like LECNN because we believe that the development of hardware devices will greatly improve inference speed. Overall, DDF&SE-DDRNet achieved the highest segmentation accuracy while also achieving satisfactory inference speed.

### 4.4. Ablation Studies

The proposed DDF&SE-DDRNet contains two ablation factors, namely squeeze and excite visual attention module and decoupled dynamic convolution. In order to verify the effectiveness of the two ablation factors, the ablation study was performed on the Cityscapes dataset using the following methods: (1) Use DDRNet-23 network structure as baseline network. (2) Only the standard convolution in the baseline is replaced by decoupled dynamic convolution. (3) Only add the SE-Module to the baseline network and record the segmentation effect of adding the SE-Module at different positions of the baseline network. (4) The segmentation results of the above methods are compared with the segmentation effects of the proposed DDF&SE-DDRNet.

The results of the ablation study are shown in Table 2, where √1–3 indicates that SE-Module is added to the first, second, and third stages of the baseline network. √4–5 indicates that SE-Module is added to the fourth and fifth stages of the baseline network. √1–5 means that the SE-Module is added to all stages of the baseline network. The symbol “-” in Table 2 indicates that there is no DDF or SE-Module present in the network.

It can be seen from the results in Table 2 that the two ablation factors in the model can effectively improve the performance of the segmentation network. Moreover, the experimental results show that the network segmentation effect is the best when SE-Modules are used in all stages of the baseline network. That is, among all the models listed in Table 2, DDF&SE-DDRNet is the optimal network model. The results of isolation experiments demonstrate that both DDF and SE-Module in DDF&SE-DDRNet contribute to improving segmentation accuracy.

### 4.5. Visualized Road Scene Segmentation Results

**Figure 4:**
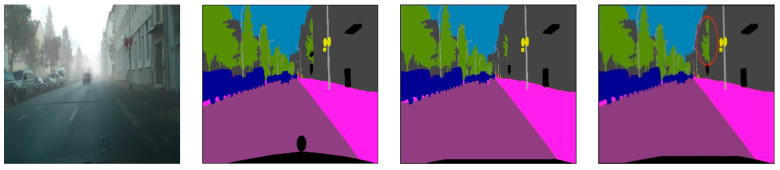

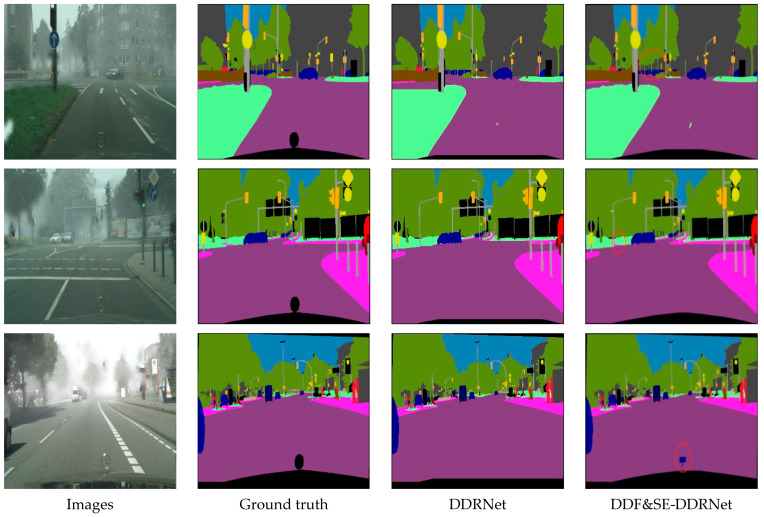
Visualized Segmentation results of three networks.

Figure 4 shows the visualized segmentation results obtained by using DDRNet and DDF&SE-DDRNet for road scene segmentation. It can be seen from Figure 4 that compared with the original DDRNet, the segmented images obtained by the DDF&SE-DDRNet-based road scene segmentation method have higher quality and finer segmentation details. For the convenience of observation, we marked the notable parts in the segmentation results output by DDF&SE-DDRNet with red circles.

The lighting in the first road scene picture in Figure 4 is uneven. The third and fourth columns in the first row of Figure 4 are the segmentation map output by DDRNet and the segmentation map output by DDF&SE-DDRNet, respectively. For the buildings and trees on the right side of the input image, the segmentation map output by DDF&SE-DDRNet reflects more details. The second to third pictures to be segmented in Figure 4 have rain or fog, respectively. The segmented images output by DDF&SE-DDRNet contain more details than those from DDRNet. The segmentation image in row 2, column 3 has a more complete outline of the trees in front of the road to the left. The segmented image in row 3, column 3, contains more detailed information on the road and grass below the traffic lights. The segmented image in row 4 and column 3 contains the Mercedes-Benz logo, while the segmented image in column 2 does not contain the Mercedes-Benz logo.

## 5. Conclusions

In this paper, we propose a DDF&SE-DDRNet network for road scene segmentation. The SE-Module in the proposed network makes DDF&SE-DDRNet robust to local disturbances in images. At the same time, DDR requires less computation than ordinary convolution, so the number of parameters required for DDF&SE-DDRNet is less than that of the original DDRNet. The experimental results on the Cityscapes dataset demonstrate that DDF&SE-DDRNet has better segmentation accuracy and inferring speed. The results of ablation studies have shown that both DDF and SE-Module in DDF&SE-DDRNet contribute to improving the segmentation accuracy, despite the fact that the segmentation accuracy of DDF&SE-DDRNet is higher than that of LECNN. However, the inference speed of LECNN is much higher than that of DDF&SE-DDRNet. We plan to reduce the number of parameters and improve inference speed in subsequent research concerning DDF&SE-DDRNet.

## Figures and Tables

**Table 1:** Segmentation results on Cityscapes dataset.

Method	PA (%)	mPA (%)	Miou (%)	FPS	Parameter
LECNN	-	-	65.9	106	0.64 M
BiSeNet V2	-	-	75.8	47	49 M
ResNet-50	-	-	76	40	25.6 M
DDRNet	96.1	85.1	77.2	51	20.3 M
DDF&SE-DDRNet	96.4	86.2	79.2	60	18.6 M

**Table 2:** Results of Ablation study.

Method	DDF	SE-Module	mIou (%)	Parameter
DDRNet	-	-	77.20	20.3 M
DDF-DDRNet	√	-	78.52	18.4 M
SE-DDRNet1	-	√1–3	77.52	20.4 M
SE-DDRNet2	-	√4–5	77.63	20.4 M
SE-DDRNet3	-	√1–5	77.92	20.6 M
DDF&SE-DDRNet	√	√1–5	79.20	18.6 M

## Data Availability

Not applicable.

## References

[1] Xu J., Park S.H., Zhang X., Hu J. (2022). The improvement of road driving safety guided by visual inattentional blindness. IEEE Trans. Intell. Transp. Syst..

[2] Chen J., Wang Q., Peng W., Xu H., Li X., Xu W. (2022). Disparity-Based multiscale fusion network for transportation detection. IEEE Trans. Intell. Transp. Syst..

[3] Xiong S., Li B., Zhu S. (2023). DCGNN: A single-stage 3D object detection network based on density clustering and graph neural network. Complex Intell. Syst..

[4] Redmon J., Farhadi A. (2018). YOLOv3: An incremental improvement. arXiv.

[5] Chen J., Wang Q., Cheng H.H., Peng W., Xu W. (2022). A review of vision-based traffic semantic understanding in ITSs. IEEE Trans. Intell. Transp. Syst..

[6] Ren X., Ahmad S., Zhang L., Xiang L., Nie D., Yang F., Wang Q., Shen D. (2020). Task decomposition and synchronization for semantic biomedical image segmentation. IEEE Trans. Image Process..

[7] Jing L., Chen Y., Tian Y. (2019). Coarse-to-fine semantic segmentation from image-level labels. IEEE Trans. Image Process..

[8] Cira C.-I., Kada M., Manso-Callejo M.-Á., Alcarria R., Bordel Sanchez B. (2022). Improving road surface area extraction via semantic segmentation with conditional generative learning for deep inpainting operations. ISPRS Int. J. Geo-Inf..

[9] Long J., Shelhamer E., Darrell T. Fully convolutional networks for semantic segmentation. IEEE Trans. Proceedings of the IEEE Conference on Computer Vision and Pattern Recognition.

[10] Yu C., Wang J., Peng C., Gao C., Yu G., Sang N. Bisenet: Bilateral segmentation network for real-time semantic segmentation. Proceedings of the European Conference on Computer Vision (ECCV).

[11] Du L., Zhang Y., Liu B., Yan H. An Urban Road Semantic Segmentation Method Based on Bilateral Segmentation Network. Proceedings of the 2023 3rd International Conference on Neural Networks, Information and Communication Engineering (NNICE).

[12] Kherraki A., Maqbool M., El Ouazzani R. Lightweight and Efficient Convolutional Neural Network for Road Scene Semantic Segmentation. Proceedings of the 2022 IEEE 18th International Conference on Intelligent Computer Communication and Processing (ICCP).

[13] Romera E., Alvarez J.M., Bergasa L.M., Arroyo R. Efficient convNet for real-time semantic segmentation. Proceedings of the 2017 IEEE Intelligent Vehicles Symposium (IV).

[14] Badrinarayanan V., Handa A., Cipolla R. (2015). SegNet: A deep convolutional encoder-decoder architecture for mage segmentation. arXiv.

[15] Hong Y., Pan H., Sun W., Jia Y. (2021). Deep dual-resolution networks for real-time and accurate semantic segmentation of road scenes. arXiv.

[16] Zhou J., Jampani V., Pi Z., Liu Q., Yang M.-H. Decoupled dynamic filter networks. Proceedings of the IEEE/CVF Conference on Computer Vision and Pattern Recognition.

[17] Hu J., Shen L., Sun G. Squeeze-and-excitation networks. Proceedings of the IEEE Conference on Computer Vision and Pattern Recognition.

[18] Li B., Lu Y., Pang W., Xu H. (2023). Image Colorization using CycleGAN with semantic and spatial rationality. Multimed. Tools Appl..

[19] Cordts M., Omran M., Ramos S., Rehfeld T., Enzweiler M., Benenson R., Franke U., Roth S., Schiele B. The cityscapes dataset for semantic urban scene understanding. Proceedings of the IEEE Conference on Computer Vision and Pattern Recognition.

[20] Yu C., Gao C., Wang J., Yu G., Shen C., Sang N. (2021). Bisenet v2: Bilateral network with guided aggregation for real-time semantic segmentation. Int. J. Comput. Vis..

[21] He K., Zhang X., Ren S., Sun J. Deep residual learning for image recognition. Proceedings of the IEEE Conference on Computer Vision and Pattern Recognition.

